# Editorial: Methods and applications in neurodegeneration

**DOI:** 10.3389/fnins.2024.1455063

**Published:** 2024-09-06

**Authors:** Jiaman Xu, Tingyuan Lang

**Affiliations:** ^1^Reproductive Medicine Center, The First Affiliated Hospital of Chongqing Medical University, Chongqing, China; ^2^Department of Neurology, The First Affiliated Hospital of Chongqing Medical University, Chongqing, China

**Keywords:** neurodegeneration, neurodegenerative diseases, method, application, model

## 1 New experimental animal models of neurodegeneration

Given that positive results obtained from current animal models are often not replicated in the clinical setting, new experimental animals for neurodegenerative diseases are urgently needed. Sanfeliu et al. analyzed the known and new neurotoxicity-induced models of neurodegeneration that may complement genetically modified models, including models based on dopaminergic (methyl-4-phenyl-1,2,3,6-tetrahydropyridine (MPTP), rotenone, and 6-hydroxydopamine (6-OHDA) models of Parkinson's disease), cholinergic (scopolamine model of Alzheimer's disease), and neuroinflammation-induced (lipopolysaccharide (LPS), streptozotocin (STZ), monomeric C-reactive protein (mCRP) models of Alzheimer's disease) neurodegeneration. In addition to rodent models, Omar et al. improved the parameters for the MPTP-induced zebrafish model.

## 2 Technical and mechanistic breakthroughs in molecular research

Single-cell technology provides the opportunity to detect heterogeneity and spatial information among individual cells, but modifying parameters based on the specific specimens remains a challenge. Hahn et al. developed a novel protocol to protect RNA quality for spatial transcriptomics while improving immunofluorescent staining quality. Also featured in this Research Topic is the work of Liu et al., who examined new clues to the neurodegeneration of amyotrophic lateral sclerosis (ALS) by employing integrative genetic and single-cell RNA sequencing. Limited protocols have been developed to isolate and analyze amyloid β-protein (Aβ) from the postmortem brain. Hong et al. have developed three distinct methods for studying Aβ from human cortical tissue. These innovative protocols and findings will undoubtedly facilitate future neuroscience research.

## 3 Novel clinical features in neurodegenerative diseases

The development of novel clinical features plays a fundamental role in the prevention, diagnosis, treatment, and mechanistic study of diseases. Xue et al. highlighted the causal link between Alzheimer's disease and type 2 diabetes mellitus. Wang et al. conducted a neutral image recognition memory task and identified novel features of event-related potentials in patients with Alzheimer's disease. Kuang et al. investigated the hypertension score, Montreal Cognitive Assessment score, and body mass index as important clinical features in patients with white matter hyperintensity lesions (WMHL). Li et al. identified potential risk factors for limb weakness in patients with herpes zoster.

## 4 Comparison of therapeutic options

Overactive bladder (OAB) is a serious and most common complication in patients with multiple sclerosis (MS). The study conducted by Majdinasab et al. showed that both solifenacin (SS) and posterior tibial nerve stimulation (PTNS) were effective in improving symptoms, while patients treated with SS had a better experience, in terms of daytime frequency, urinary, incontinence, and treatment satisfaction.

## 5 Conclusion

These researchers highlighted the latest findings in neurodegeneration, including experimental animal models, technical and mechanistic breakthroughs, clinical features, and therapeutic options. These innovative studies, comprising the Research Topic Methods and Application in Neurodegeneration, represent fundamental and impactful recommendations for future basic, clinical, and translational studies in neurodegeneration-related diseases.

